# Calming Effects of Touch in Human, Animal, and Robotic Interaction—Scientific State-of-the-Art and Technical Advances

**DOI:** 10.3389/fpsyt.2020.555058

**Published:** 2020-11-04

**Authors:** Monika Eckstein, Ilshat Mamaev, Beate Ditzen, Uta Sailer

**Affiliations:** ^1^Institute of Medical Psychology, University Hospital Heidelberg, and Ruprecht-Karls University Heidelberg, Heidelberg, Germany; ^2^Institute for Anthropomatics and Robotics, Karlsruhe Institute of Technology, Karlsruhe, Germany; ^3^Department of Behavioural Medicine, Faculty of Medicine, Institute of Basic Medical Sciences, University of Oslo, Oslo, Norway

**Keywords:** safety signal, stress axis, cortisol, oxytocin, amygdala, C-tactile, HRI (human robot interaction), heart rate variability

## Abstract

Small everyday gestures such as a tap on the shoulder can affect the way humans feel and act. Touch can have a calming effect and alter the way stress is handled, thereby promoting mental and physical health. Due to current technical advances and the growing role of intelligent robots in households and healthcare, recent research also addressed the potential of robotic touch for stress reduction. In addition, touch by non-human agents such as animals or inanimate objects may have a calming effect. This conceptual article will review a selection of the most relevant studies reporting the physiological, hormonal, neural, and subjective effects of touch on stress, arousal, and negative affect. Robotic systems capable of non-social touch will be assessed together with control strategies and sensor technologies. Parallels and differences of human-to-human touch and human-to-non-human touch will be discussed. We propose that, under appropriate conditions, touch can act as (social) signal for safety, even when the interaction partner is an animal or a machine. We will also outline potential directions for future research and clinical relevance. Thereby, this review can provide a foundation for further investigations into the beneficial contribution of touch by different agents to regulate negative affect and arousal in humans.

## Introduction

The tactile sense is one of the first that a human develops. A newborn child has the first contact with its environment, such as its clothes or its cradle. In particular, touch by the parents has been proposed to be important for development, e.g., feeling their touches on its skin, but also by feeling tactile input when it actively moves toward them, with an important impact on the child's development (1, 2).

Even in adulthood, being touched and touching others is a central element of social interaction and social relationships (3). It has been suggested that social touch is one mechanism for beneficial health effects of social relationships. The effects of positive social interaction, in general, show effect sizes equaling or exceeding those of well-established behavioral factors, such as smoking cessation or sports (4); some of them might be due to touch or intimacy. Touch has also been ascribed as important functions during bonding [e.g., (5)], communication (6–8), and reward [e.g., (9, 10)].

It has often been proposed that social touch can buffer stress and has calming effects [e.g., see overviews by Burleson and Davis (11) and Morrison (12)], but the underlying preconditions and mechanisms of this positive effect are not sufficiently investigated yet. Several studies show a reduction of psychobiological fear or stress responses in neuro-physiological and endocrine outcomes after touch [e.g., (13–15)]. Here we would like to put forward the possible mechanism that touch acts as a social signal for safety, which communicates to the receiver that “things are ok,” and thereby inhibits fear and stress responses. The assumed neural processes in terms of responses to touch signals and their mediation of attenuated fear and stress responses will be outlined below.

Of course, touch can also occur as an act of aggression or in order to threaten an interaction partner. In these negative interaction situations, both the expectations and the physical properties are different (6, 7, 16), with violence as an extreme form of touch and physical pain as a potential consequence. There is surprisingly little research on the stress-inducing effects of touch. For example, during a physical examination, the medical doctor's announcement of pain may induce stronger pain than the touch itself; this is mostly investigated in the context of placebo- and nocebo-research (17). However, in order to determine the potential beneficial effects of touch by agents other than humans, it is crucial to also evaluate whether and when it can be experienced as negative.

The increased use of intelligent robots as service machines, especially in the medical context, makes human–robot interactions more and more frequent in daily routine as well as in healthcare. This raises the question of whether the beneficial effects of touch depend on the social source of the tactile stimulation or whether they can also be elicited by mechanical or robotic devices. This question is also generally important in medical situations since most humans experience illness, physical examinations, and surgery as threatening. Therefore, robots interacting with humans in a way that supports mental and physical well-being have the potential for directly supporting individuals at risk and also the healthcare system in general. With the current demographic development, more and more people, including the elderly, also live alone. At the same time, when deprived of social touch, e.g., lonely persons or patients in self-isolation or quarantine, humans show higher levels of stress and more symptoms of mood, and anxiety disorders (18). This poses the question of whether an absence of human touch can be (partly) compensated for by an animal companion or a machine. In many of the studies on gentle touch perception, the stimulation is performed by a machine and is evaluated as similarly pleasant by healthy participants than when performed with the hand (19). This suggests that touch by actors other than humans can give rise to comparable hedonic experiences.

The goal of this conceptual review is to give an overview of experimental research on the calming effects of touch, taking into account different interaction partners. In the following discussion, the evidence for stress-reducing effects of touch by humans, animals, and even robotic machines that might be of relevance for clinical contexts will be summarized. Supporting the view of at least partly comparable effects, we propose joint underlying neurobiological mechanisms. These will be outlined in the following section.

### Neural Mechanisms Underlying the Calming Effects of Touch

**Figure 1:**
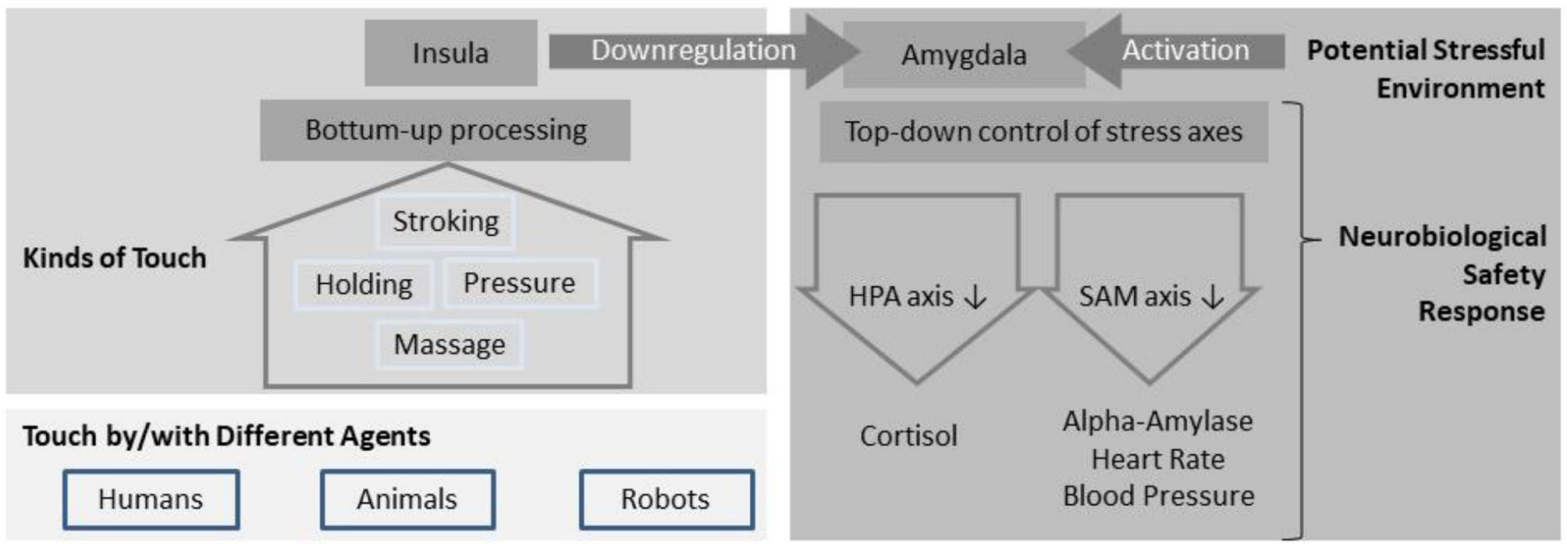
Mechanisms of touch potentially acting as safety signal: In a dangerous environment, the amygdala is activated in order to allow for rapid reactions via the stress axes (e.g., fight or flight response). Tactile perception from different social and non-social contexts is processed in the insular cortex, which has a regulating influence on the amygdala and can therefore dampen the stress response. A calming or relaxing effect of touch might therefore be based on signaling safety (absence of danger) on a neurobiological level.

In the following paragraph, we will describe two possible neural circuitries which might mediate touch acting as safety signal: inhibition of the amygdalar fear response *via* the posterior insula and activation of the reward system for facilitating approach behavior. In the latter, stress dampening effects are assumed to be less dominant. Both the bottom-up processing of the tactile experience and the top-down regulation of the fear/stress response are displayed in Figure 1.

#### Regulation of Fear and Stress Responses

The state of literature on fear inhibition describes a down-regulation of amygdala activity through the input of the prefrontal cortex (PFC) and the insula. Evidence suggests that, in particular, the pathway *via* the insula is also involved in the stress-reducing effects of touch.

The amygdala is widely known as the neural center of fear, although being involved in various other functions (20, 21). Mostly animal literature, but also human studies, shows that the amygdala is not a homologous structure but is composed of subnuclei with different functions. The lateral amygdala, the primary sensory input site, and the basal amygdala (together BLA) are involved in fear learning, while its central nucleus (CeA) is involved in the expression of fear (22). The expression of fear results in the activation of two stress axes (see “Section Neuroendocrine mediators and stress response” below) for a flight or fight response, which is measurable in endocrine or psychophysiological outcomes.

The amygdalar neurons within the subnuclei are under the inhibitory control of local GABAergic interneurons (23) and the medial intercalated neurons (24). Control from other brain regions comes from the infralimbic ventromedial prefrontal cortex (25) and the insula (26, 27). The inhibitory control of the amygdala *via* the PFC and the insula, together with the amygdala, is a network for top-down and bottom-up emotion generation. Bottom-up processes describe the information flow starting from the stimulation of specific receptors to subsequent neural reactions. Top-down processes describe, e.g., modulating influences from the PFC (associated with cognitive influences such as appraisal or evaluation) on the perception and processing of touch.

The posterior insula, termed sensory insula, exhibits convergent responses to simultaneous multisensory stimulation (28) and has afferent intracortical and thalamocortical as well as efferent amygdala connections (26, 29). The insula is therefore well-suited to modulate amygdala activation based on both top-down and bottom-up input. Such input could be triggered from a particular “all-clear” signal or, in other words, a safety signal.

#### Neural Processing of Safety Signals

Few studies have investigated the neural response to—mostly visual—safety signals and point to an involvement of both the posterior insular cortex and the striatal reward system in the processing of visual safety cues. Safety signals, in general, have been first described by (30) as one form of internal inhibition of conditioned reflexes, with a former neutral stimulus predicting the non-occurrence of an aversive event after a learning process. It can be assumed that social stimuli such as social touch can not only be learned to predict safety but also to have the property to “prepare for safety,” analogous to some stimulus types that include preparedness for fear (31). Everyday life examples for safety signals would be, e.g., a calm voice and also the face of the romantic partner (32).

Based on the findings on inhibition of fear, Kong et al. (33) have proposed a regulatory model stating that the posterior sensory insula projects to BLA which orchestrates CeA and bed nucleus of striatum terminals that subsequently mediate the behavioral output in response to a safety signal. This is supported by animal studies showing that a knock-out of the posterior insula leads to deficient inhibition of fear (27, 34).

On the other hand, very early work by Dickinson and Pearce (35) suggested a further mechanism by which safety signals could act. These authors suggested that safety signals inhibit the aversive system while at the same time disinhibiting the appetitive system. Safety signals would thereby facilitate approach behavior and act as reinforcers. These possible rewarding effects of safety signals led Pollak et al. (36) to suggest them as “behavioral antidepressants.” Other studies supporting this idea showed that a safety signal increased the slope and the amplitude of conditioned stimulus-evoked field potentials in the caudatoputamen (37), reduced the activity in the amygdala, and increased the activity in the striatum (38). The reward system itself has regulatory influences on the stress axes [e.g., (39, 40)]. This raises the possibility that safety signals activate the reward system which then downregulates amygdala activation. Thus, touch as a safety signal might also execute its stress-inducing effect *via* reward system activation.

#### Neural Processing of Tactile Information

Several human imaging studies revealed that touch activates a broad neurocircuitry including the insula, orbitofrontal cortex, and anterior cingulate cortex (41–43).

An especially “social” experience of touch has been described as being conveyed by low-threshold unmyelinated peripheral afferent fibers [C tactile (CT) fibers]. These fibers respond preferentially to gentle, slow, caress-like stroking at skin temperature (44), and their activation is generally perceived as pleasant (45). CT afferents project to the posterior insula (46, 47). For instance, Gordon et al. (48) showed that CT–targeted affective touch to the arm activated the insula and the mPFC/dorsal–anterior cingulate cortex (dACC). Lesions of the insula in turn, impair the perception of affective touch (49). Recently, the insula was shown to be also activated by A-beta afferents (50). Thus, bottom-up input from different mechanoreceptors reaching the insula has the potential to dampen the stress response. Connectivity analyses with a mPFC/dACC seed revealed co-activation with the left insula and amygdala. These studies therefore suggest regulation of the amygdala by touch acting as safety signal *via* mediation of the insula.

In addition to bottom-up influences, top-town influences have also been discussed, for example, expectations. This influence can affect the valence of the touch perception from prefrontal and limbic regions (51). It remains still to be determined how bottom-up and top-down influences on touch processing interact to modulate the stress response. The involvement of the reward system in this process also needs to be clarified. One region coding for the reinforcing aspect of touch is the ventral striatum (52). Our own work has shown a joint activation of the insula, putamen, and caudate (53) during CT-targeted touch. This involvement of the striatum may point to the second neural mechanism of touch acting as safety stimuli *via* the reward system. However, striatum activation is only found occasionally in studies on pleasant touch, so more evidence is needed.

Taken together, inhibition of amygdala fear *via* the insula is a highly plausible underlying mechanism of touch acting as safety signal. Potentially, amygdala inhibition is furthermore due to reward system projections. Since research on safety signal processing is limited to visual signals so far, it has to be investigated yet whether tactile safety signals act on the same processes. On the other hand, opposite mechanisms may account for the stress-inducing effects of touch in negative contexts *via* increasing the amygdalar responses, yet this remains to be investigated as well.

### Neuroendocrine Mediators and Stress Response

In addition to neuroanatomical connections, mediating neuromodulators, and neurotransmitters such as oxytocin and dopamine released in response to touch may be regulating the above-mentioned limbic and reward areas (54–56). Especially oxytocin has been shown to be released during intimate touch (57–60), while dampening stress and fear (61, 62). Administered exogenously, oxytocin increases the neural and subjective response to touch (15, 56). Histological investigations show a high density of oxytocin receptors in the human insula, striatum, and amygdala (63), which constitutes an additional regulatory mechanism of the neural circuitry mentioned above.

#### Stress Axes

The fear and stress response triggered by the amygdala reaches the periphery by two main axes of stress hormones, the hypothalamic–pituitary–adrenocortical (HPA) axis (64) and the sympathetic–adrenomedullary (SAM) system [(65); see also Figure 1]. HPA axis responses are mediated through a cascade of hormones from the central nervous system (corticotrophin-releasing factor), which then stimulate adrenocorticotropic hormone and cortisol secretion in the periphery. As dynamic negative feedback of the HPA axis, the increase of cortisol will—*via* the activation of mineralocorticoid and glucocorticoid receptors—reduce further activation and, in turn, initiate the recovery from stress (66). Cortisol in saliva is one established key marker for assessing stress levels (67). The SAM system, on the other hand, facilitates a fast reaction to acute threat *via* the adrenal medulla releasing catecholamines. The parasympathetic component of the SAM influences, e.g., the heart rate (HR) *via* the vagus nerve or salivary alpha-amylase as a product of beta-adrenergic activity (68). The heart rate variability (HRV) is an established marker for a healthy adaptation to stress (69) that is regulated by the autonomic nervous system, both by its parasympathetic branch that is known for the “fight or flight response” and its parasympathetic branch.

Taken together, touch has the potential to exert a calming and stress-dampening effect *via* these neurobiological mechanisms. Indeed the findings from many studies suggest that such an effect might be observed across a variety of different contexts due to a joint phylogenetic basis. We assume that the evolutionary circuitries underlying touch as a safety signal are activated through all kinds of touch, yet context and personal factors can moderate the effects. In order to systematically explore these effects, we performed a literature search and will summarize the findings in the consecutive sections for the different contexts.

## Methodological Overview

With a focus on basic research, we chose to include studies in healthy human adults published in English. We searched the platforms Pubmed.gov, Web of Science, and Google Scholar with the search terms “touch,” “massage,” “stress,” “fear,” “cortisol,” “heart rate,” “arousal,” “blood pressure,” “animal,” “pet,” “machine,” “physical contact,” “tactile,” among others, individually or in combination. Boolean operators were used to search with multiple terms. Further papers were retrieved from the reference lists of papers found this way. Given the large number of results, we decided at this point to set up further exclusion criteria and to only include studies that fulfilled the following criteria: (1) outcomes were measured in adult humans (not infants), (2) measures of stress, anxiety (subjective and/or physiological), or negative affect were used, and (3) the extent, type, and duration of tactile contact was explicitly stated. This excluded studies, for example, where the information was restricted to the statement that the participants “interacted” with a (robot) animal without it being clear whether this included touch. Both self-initiated touch situations (active touch) and other-initiated touch situations (where the human receives passive touch) are discussed. Based on these criteria, the following sections “Human-Human Touch”, “Touch between Human and Animal”, and “Touch between humans and artificial object” will give a summary on the most relevant experimental studies reporting physiological, hormonal, neural, and subjective indicators of the positive role of touch in different contexts on stress, arousal, and negative affect.

## Human–Human Touch

When analyzing human-to-human touch, behaviors as listed in Figure 1 (i.e., stroking, holding, pressure, massage) can be interpreted. In addition, in 2018, Lee Masson and Op de Beeck (70) published a socio-affective touch expression database, based on video sequences, to be rated on the dimensions naturalness and valence. This database can help in structuring human-to-human touch experiences but has not systematically been tested with regard to different relationship types. Being touched by another human can yield substantially different responses depending on the personal relationship. In a study investigating touch between close friends, Kawamichi et al. (71) found that the participants evaluated hand-holding with a close female friend as more relaxing than holding a rubber hand and showed parallel dampening effects on neural activation when processing aversive visual stimuli while in an fMRI scanner. In order to account for the effects of personal relationships between the persons touching and being touched, we will summarize studies separately for different relationship forms (romantic, professional). Apart from parent–infant touch (which is not in the scope of this review), human touch studies focused mostly on touch between adult romantic couples and on touch in a medical context, particularly the effects of massage.

### Touch Between Romantic Partners

Studies on touch between romantic partners suggest that affective touch can reduce subjective and psychobiological stress levels during standard stress in the laboratory (13, 72) and in couples' everyday life (73, 74). Couples who reported more physical intimacy in everyday life had lower cortisol levels on a momentary basis (73) and higher oxytocin levels in plasma at baseline before a lab stress test (75). In another study, higher levels of non-verbal affection in intimate relationships (parents, partner) were associated with lower HR and blood pressure levels (76). In a functional MRI study, Coan et al. (77) found that hand-holding—the partner's hand in particular—during the anticipation of pain reduced unpleasantness and bodily arousal as well as the neural threat response in *N* = 17 women. In another study, pupil dilation during the Stroop test was interpreted as an arousal marker, and study participants who held hands with their partner showed accelerated habituation to stress and less pupil reactivity (although this was not a tonic pupil response) than those in the non-hand-holding condition (78). Being stroked by the partner also decreased HR, and the decrease was related to the quality of the relationship (14). Furthermore, 10-min hand-holding with the partner while watching a romantic video reduced subsequent blood pressure during public speaking (79). Overall, affective touch between partners can reduce stress levels and psychobiological stress reactivity as measured with different markers of arousal. Of note, however, is that, so far, affective touch between romantic partners has not been related to the duration of the relationship. During the beginning of a romantic relationship, overall increased stress and arousal have been found (80). Based on this, it might be assumed that touch during the beginning of an erotic or intimate relationship would rather increase arousal and psychobiological stress levels than reduce stress.

Beyond this, touch not only serves as a calming agent but can also communicate specific emotions (6) and thereby even serve to communicate anxiety or aggression (45). So far, we are not aware of systematic research on the effects of positive affective touch in comparison to aggressive touch or physical violence in intimate relationships. It could be assumed that touch might serve as an intensifying factor of both bonding and affiliative behavior on the one side and anxiety and stress on the other side, thereby acting either as a safety or a threat signal.

### Touch in Professional Relationships

Studies on non-romantic human touch have used both highly controlled standardized touch movements and also static holding/ hugging or complex massages [e.g., Thai massages; (81)].

Using such a standardized design in an early study and with a small sample size only, an experimenter touched the wrist of *N* = 8 healthy subjects for 30 s (82). This led to a decrease in HR, indicating relaxation. Touching the wrist by the subject him/herself with their other hand did not decrease HR. A similar effect of 60-s wrist-holding by an experimenter also occurred when the subjects (*N* = 20) were confronted with a cold pressor stressor (83). HR was also reduced by 5 min of CT-touch (N = 29) (10), as well as skin conductance response (*N* = 34) as a measure for unspecific arousal (84).

In a within-subject design, von Mohr et al. (85) compared different stroke frequencies and found that the partner's slow touch (in comparison to fast touch) reduced pain levels to standard pain in the laboratory. This data was in line with earlier results from the same group (however, not in couples) that slow affective touch reduced feelings of social exclusion during the Cyberball task (86). In a patient sample (*N* = 29 individuals with coronary illness), different kinds of touch led also to reduced HR and lower blood pressure (87). Taken together, these studies indicate a regulatory influence of simple static touch on autonomous nervous system activity.

#### Massage Studies

Classical Western or also traditional Eastern massage usually involves large parts of the body and is combined with treatments such as aroma oils or relaxing music. Therefore, the effects of music, odors, and oils are often not clearly separable from the effects of the touch itself. In addition, massage touches not only the skin and stimulates the tactile system but also the deeper tissue and muscles, which might also account for some of the beneficial effects on well-being. As all these effects cannot be disentangled from the sole effect of touch, we only refer to few exemplary studies in the following discussion. The effects of massage on stress relief become evident in patients with various conditions.

A 7-min standardized hand massage by an unknown experimenter led to a decrease in cortisol levels in 29 healthy volunteers as compared to simply holding an object in their hand while the experimenter was present (88). In a subgroup of highly self-critical individuals, the hand massage additionally decreased alpha-amylase levels. Likewise, receiving a 5-min hand massage reduced subjective stress, anxiety, and fatigue in *N* = 40 healthcare professionals (89). In palliative care patients, salivary chromogranin A, as another biomarker for stress by SAM activation, was reduced after a hand massage as well (90). When waiting for ambulatory surgery, a 5-min hand massage reduced anxiety in *N* = 45 patients as compared to controls without a medical intervention pending (91). This finding indicates a function of safety especially in the presence of acute threat.

A classical (whole body) massage for 30 min reduced cortisol and subjective stress levels in 34 breast cancer patients (92). Patients (*N* = 24) suffering from back pain receiving two 30-min sessions of massage therapy reported experiencing less pain and anxiety and showed higher serotonin and dopamine levels than controls in a relaxation intervention (93). On the other hand, actively giving a massage also shows stress-dampening effects: elderly retired volunteers showed lower anxiety scores, salivary cortisol, and long-term catecholamine levels after giving a standard massage to infants in a hospital (94).

Taken together, these studies indicate a potential positive effect of not only being massaged but also of giving massages on subjective stress and neuroendocrine response, yet these have to be interpreted with caution due to the multifaceted uses of touch.

#### Pressure

An osteopathic technique called deep touch, using larger pressure of 44 N toward the rear head muscles for 90 s, led to an increase in HRV in *N* = 35 healthy participants (95). A deep hands-and-feet massage with pressure of about 2.5 N and a velocity of 1–5 cm/s for 80 min in 63 volunteers, on the other hand, led to a decrease in HRV and HR, together with a reduction in cortisol and insulin levels (96). In a study with 15 min of light and moderate pressure massage in *N* = 20 (97), the participants who received the moderate pressure massage exhibited a parasympathetic nervous system response characterized by an increase in high frequency (HF), suggesting increased vagal efferent activity, and a decrease in the low frequency/high frequency (LF/HF) ratio, suggesting a shift from sympathetic to parasympathetic activity that peaked during the first half of the massage period. On the other hand, those who received the light pressure massage exhibited a sympathetic nervous system response characterized by decreased HF and increased LF/HF. Therefore, pressure also seems to regulate the autonomous stress axes.

## Touch Between Human and Animal

Touch with an animal—trained or untrained—is an element of animal-assisted therapy, a non-pharmacological intervention aimed to improve human health in a wide range of conditions and patients. This type of therapy has become more and more popular for clinical conditions such as dementia, depression, and post-traumatic stress disorder, among others. Whereas studies appear to point at the beneficial effects of animal-assisted therapy for many health outcomes, they often address parameters other than stress reduction and, in part, suffer from methodological problems [e.g., as reviewed in Charry-Sánchez et al. (98)]. In the following discussion, we will focus on summarizing experimental studies meeting more rigid criteria with regard to the variation of the touch stimulus and the outcomes.

In the studies meeting our criteria, the animal of choice was usually the dog. In one such early study, HR and blood pressure were collected in 60 participants during different types of interaction with a dog (tactile, verbal–tactile, conversation in the presence and the absence of a dog, and rest) which each lasted for 6 min (99). For the tactile condition, the participants were instructed to fondle and pat the dog or let it sit on the lap while refraining from talking to it. Blood pressure was lower in the tactile and rest condition than during the verbal and verbal–tactile condition. Blood pressure was also higher during the conversation than during all other conditions. Thus, patting the dog and resting appear to have had similar effects, with no clear advantage of touch.

In a related study, 10 dog owners and 10 controls participated (100). The dog owners sat in a chair and petted, stroked, and talked to their dog for 3 min, whereas the controls just sat there. Levels of cortisol and HR to measure activation of the autonomic nervous system were assessed during the interaction/sitting still and the subsequent 57 min. In addition, insulin was measured to reflect vagal nerve tone and oxytocin to investigate the interaction's effect on stress and arousal. The cortisol and the insulin levels decreased in both groups, whereas HR only decreased in dog owners. At the same time, the dog owners' oxytocin levels increased shortly after the interaction. Thus, the decreased HR in dog owners could have been due to the touch itself or due to bonding with their dog. As cortisol also decreased in the group sitting still without a dog, the study only provides weak evidence for a specific beneficial effect of a dog on the stress response.

Whereas, the majority of studies was performed with a dog as touch target, there is also one study with a horse. HR and subjective arousal were measured in 18 participants before, during, and after stroking a horse for 90 s (101). HR was highest during the first 10 s of stroking and decreased steadily across the remaining time. Subjective arousal decreased as well, and tiredness increased. However, as no control condition was administered, it is not known whether the HR changes were specific to the stroking. Therefore, evidence of stress-dampening effects of touching an animal is not very strong in these studies so far.

### Comparing Animal Touch With Quiet Reading

The role of the relationship with the dog was investigated in a study using quiet reading as a control condition (102). Here blood pressure, HR, and respirator rate were measured in 24 participants while they petted an unknown dog, a known dog, or read quietly for 9 min in three sessions. Blood pressure decreased more for petting the known dog than the unknown dog. *Post hoc* comparisons were only performed for the two dog conditions, but it appears as if the decrease in blood pressure was similar for the known dog and reading and that blood pressure was overall lowest for reading. Similarly, HR and respiratory rate appear to have been lowest for reading compared to the other two conditions where the values were rather similar. Thus, whereas petting a known dog had positive effects on arousal, quiet reading had the same calming effect.

In a similar study using an unknown dog only, blood pressure and HR were compared in 20 subjects during 11 min of reading and 18 min of petting a dog without any verbal interaction, preceded by 5 min of greeting the dog (103). Blood pressure, but not HR, was lower while petting the dog than while reading. However, since the duration of the two conditions differed by 7 min plus a “greeting period” of 5 min, it is not clear whether the change in blood pressure was due to the tactile contact with the dog or the passage of more time.

Reading aloud and quiet reading served as a control condition to petting and talking to a dog for 10 min in a study with 92 students (104). Before and afterwards, blood pressure, mean arterial pressure, HR, and state, and trait anxiety were measured. Mean arterial pressure, blood pressure, and HR were lower when petting the dog than during all other activities. State anxiety was lower for quiet reading and petting compared to the other activities. Descriptively, all these measures were lowest for quiet reading. Petting the dog had, again, no clear advantage regarding stress reduction over quiet reading. However, this does not mean that tactile interaction with a dog is ineffective, but that quiet reading as a measure of stress reduction presumably has been underestimated. It is not clear if the mechanisms underlying these effects are similar. At least the bottom-up mechanisms are different since different sensory receptors and processes are involved.

Yielding similar results, a different study measured blood pressure and several hormones, among which is cortisol, in 18 participants before and after they read quietly or interacted with one of 18 dogs (105). This interaction included talking, stroking, playing with the dog, and scratching its body and ears for 30 min. Both conditions induced similar changes in all measures, and there were no significant differences in blood pressure, levels of cortisol, phenyl acetic acid, and dopamine. All these measures decreased similarly following reading and interacting with the dog. Only beta-endorphins, oxytocin, and prolactin increased more following an interaction with the dog than during reading. This points more at bonding than on specific effects on stress relief.

Nevertheless, all these studies indicate that petting a dog, optimally one that is familiar to the touch provider, can have calming effects that become obvious in various measures. This points at the potential of dogs to act as safety signals. Studies that investigated touch effects following stress induction can provide more insight into this potential, and three more recent ones will be described in the following section.

### Touch Following Arousal Induction

One such study investigated the effect of petting a dog vs. a teddy bear on coping with a stressful situation in a large sample of 223 students (106). Blood pressure, state anxiety, and HR were assessed before and 10 and 20 min after the Trier Social Stress Test (TSST) (107). The participants had 5 min to prepare a short speech to be presented in front of a panel, followed by an arithmetic task. During both tasks, the participants were instructed to continuously pat the dog (experimental group) or a dog-size teddy bear (control group). Blood pressure was lower for all participants who had petted the dog compared to the teddy bear. State anxiety was lower for the group who had patted the dog. This effect was mainly driven by participants with high trait anxiety at the timepoint of 10 min after the TSST. HR was lower for the participants with high trait anxiety who had petted the dog compared to the teddy bear, but not for those with low trait anxiety. Thus, participants with high anxiety benefitted from touch with a living furry animal. However, it is also possible that the stuffed animal in itself already had a stress-reducing effect. This question was addressed in a different study where 58 participants were presented with a tarantula spider and told that they might be asked to hold it (108). Following this announcement, the participants split into five groups and asked to either pet a rabbit, a turtle, a toy rabbit, a toy turtle, or wait for 2 min (control group). State anxiety was measured at baseline, after stress induction, and after petting one of these objects or waiting. The participants who had petted an animal reported lower state anxiety compared to those who waited, whereas the anxiety scores of the participants who had petted a stuffed animal did not differ from the control group. The state anxiety scores following petting a real rabbit or a real turtle or soft- vs. hard-shelled animals/objects did not differ. The authors inferred that it is not the texture of the petted object or petting *per se* that lead to anxiety reduction, but only petting a living animal. Thus, this study provides evidence for the stress-reducing effects of touching a rabbit and even a turtle.

However, a further study where stress was induced by preparing and giving a speech, there was no evidence for the stress-reducing effects of petting an animal. In this study, blood pressure, HR, and state anxiety were compared in a sample of 36 participants that either kept a dog on their lap during preparation and the speech itself or not (109). While holding the dog, the experimental group was also allowed to talk to the dog and pet it. Whereas, preparing and holding the speech increased blood pressure, HR, and state anxiety, the presence of a dog did not affect these measures.

To conclude, the listed animal studies are difficult to compare due to a large variety of comparison conditions. Different animals were also used, and even the familiarity with these animals varied. Verbal interaction while petting may be a confounding factor due to the associated arousal. Measures of physiological arousal are often found to be lower during quiet reading than during interaction with an animal, but it appears difficult to draw conclusions regarding the stress-reducing effects from these setups. A better approach may be to first induce arousal and subsequently measure the effect of touch. Studies with this approach show some, however inconclusive, evidence for the stress-reducing effects of living animals compared to toy animals.

## Touch Between Humans and Artificial Object

**Figure 2:**
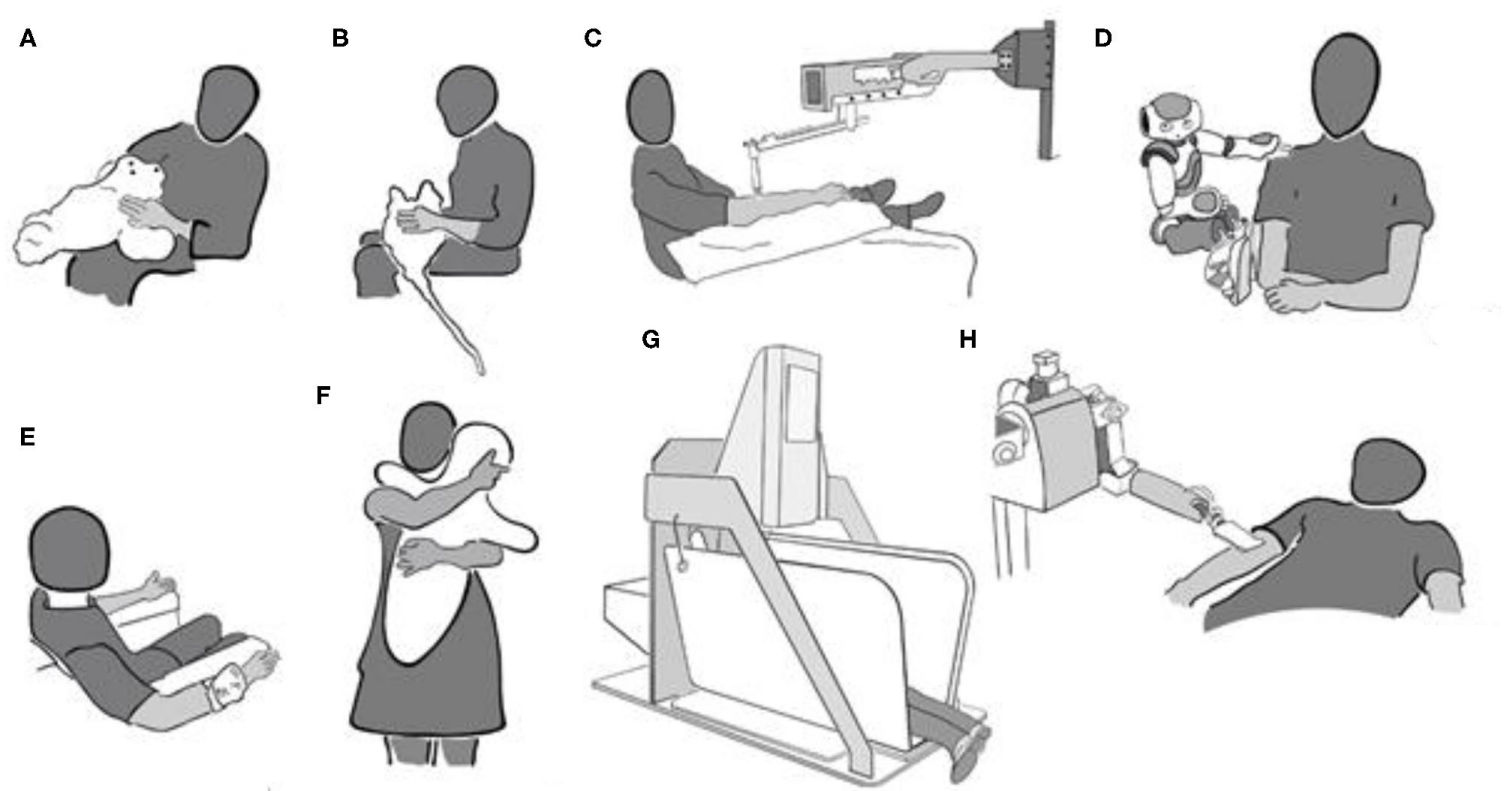
**(A–H)** Schematic overview over devices used to perform touch.

In the study of Robinson et al. (110), participants from a residential care facility interacted with and touched the robot seal “Paro” (111) for 10 min (see Figure 2A). Paro responds to visual, auditory, and tactile stimuli by moving or making small noises. Blood pressure and HR were measured before and directly after the interaction and 5 min later in 14 participants who interacted with Paro. Whereas, all these participants touched Paro during the 10 min that they interacted with him, it is not specified how much time of these 10 min was devoted to touch. Compared to a control group of seven residents who did not interact with Paro, the experimental group's systolic and diastolic blood pressure decreased from baseline, and their HR also decreased over time. Diastolic, but not systolic, blood pressure increased again 5 min after the robot had been removed. Whereas, these results are promising, the low number of participants warrants replication.

A similar kind of furry-animal like device is the “Haptic Creature” (112). The Haptic Creature (see Figure 2B) recognizes touch and responds by different forms of breathing, purring, and ear stiffness. Following a baseline where 38 healthy participants were sitting alone with the robot out of sight, the Haptic Creature was placed on their lap either turned off or while simulating the breathing of an animal (112). Galvanic skin response (GSR), respiration level, and HR were recorded during stroking and baseline, each lasting for 75 s. One hand was used to stroke the robot, and the other was kept on its side where the breathing can be felt. Subjective reports of arousal, emotional valence, and anxiety were collected after baseline and after interaction with the active or inactive furry robot in a within-subject design. When the robot was breathing compared to be inactive, respiration rate, HR, and state anxiety were lower, whereas emotional valence was more positive. Differences between baseline and interaction periods were not analyzed, but descriptive data suggest that GSR increased for both active and inactive interaction compared to baseline, whereas subjective arousal and valence were only affected by active interaction. HR and respiration rate also increased for the Haptic Creature being switched on or off, but more so when it was inactive. State anxiety also increased for the inactive robot compared to baseline, but it decreased for the active robot. Thus, it was not the mere presence of the Haptic Creature that produced relaxing effects (apart from those captured by GSR), but the fact that it was animated.

Investigating the effect of longer-lasting touch on autonomic function, Triscoli et al. (14) used a paintbrush attached to a robotic device (linear tactile stimulator; Dancer Design; St Helen's, United Kingdom) which delivers stroking at a highly replicable force (Figure 2C). The participants were stroked on their forearm with a slow CT-targeted velocity of 3 cm/s for about 35 min. This type of stimulation intends to mimic a gentle human caress, and the healthy participants rated it as similarly pleasant as touch at the same velocity performed by hand (19). HRV increased during stroking touch, but not during vibration at 100 Hz in a comparison group. This might indicate improved cardiovascular reactivity by stroking touch. At the same time, subjectively reported stress was not different following any type of touch compared to before. Cortisol levels decreased for both types of stimulation, leaving the question open on whether the changes in cortisol were due to lying still for a long time or to having been touched.

### Touch Following Arousal Induction

Several studies assessed the potential beneficial effects of touch after inducing arousal or some form of stress. In the study of (113), 67 healthy participants were touched by a “NAO” -robot while viewing movies with multiple startling scenes (see Figure 2D). GSR, HR, HRV, and respiration rate were recorded during a baseline in which a neutral movie was shown and compared to the activity during the scary movie. For eight times during the movie, the robot touched the participant on the shoulder and the upper arm for between 10 and 40 s. At the end of the touch, the robot also uttered some calming words (“Luckily, it is just a movie”). The participants in a control group watched the movie with the robot being present and moving in a similar way, but without making physical contact. Subjective ratings on different scales were collected before and after the scary movies. HR increased for the participants who did not receive touch, whereas it decreased for the participants who received touch. It appears as if there was no difference in the other measures. The subjective ratings regarding arousal and positive and negative affect were also not different. Thus, there is some evidence for arousal reduction through touch, but as touch always was combined to calming words, the respective contributions to the observed effect are not known.

In a similar experiment by the same group, additional saliva samples were collected and respiration rate was measured (114). Touch lasted here between 30 and 55 s. In this experiment, no differences between touch and the control condition were found for any of the measures (cortisol, GSR, HR, HRV, affect scores, and respiration rate). As a potential explanation for the discrepant findings in these two studies, the authors suggest that the participants in the 2019 study were already familiar with the robot before the experiment began. Getting acquainted with the robot may have promoted the stress-reducing effects by its touch. This is a plausible explanation given the differences found in animal studies between dogs known to the participant and unknown dogs.

Also using an emotional film, Cabibihan and Chauhan (115) performed a study on 30 healthy (student) participants. Ten of them received touch by their partner, 10 received no touch, and 10 received tele-touch (see Figure 2E). With this tele-touch system, pressure, and temperature information from the experimenter's hand are transmitted and presented to the participant *via* a cuff-like device as vibration, heat, and tickle. HRV and GSR were collected while the participants looked at an emotion-eliciting film. Touch was applied during the film scene that had shown the highest heart rate in pilot studies and lasted until the end of the movie (for 3 min and 29 s). HRV and GSR variations were higher in the control group than in the human and tele-touch groups. HRV for human touch and tele-touch did not differ, but GSR variation was higher for tele-touch than for human touch.

Several studies assessed the effect of “Hugvie,” a cushion with the shape of a minimalistic human (see Figure 2F) during telephone conversations with an unknown human—which may be considered an arousing situation. A sample of 18 women (mean age, 64) was split into two groups that had a 15-min conversation with a stranger either with a mobile phone (*N* = 9) or with the mobile phone placed inside Hugvie (*N* = 9). The cortisol levels were lower for the participant group who had used Hugvie (116), whereas subjective reports of calmness and positive and negative affect did not differ between the two groups. In a similar study with 29 healthy elderly participants (men and women with a mean age of 65 years), state anxiety following the conversation was lower when Hugvie had been used (117). State anxiety was lower following conversation in the group of 14 participants that had used Hugvie, but there was no difference in subjective stress and cortisol levels. In a further study, 19 participants listened to stories when they were transmitted *via* a speaker placed inside Hugvie or through a speaker in the absence of Hugvie (118). When the speaker was inside Hugvie, the participants hugged it while listening. Global field power, power in all frequency bands, and permutation entropy were lower during listening and hugging Hugvie than during listening alone and during rest. This was interpreted as indicating higher levels of relaxation when using Hugvie.

Taken together, the evidence points at some positive effects of robot interaction on stress-related measures, but only in some and not all measures. As the measures used also differed in almost every study, the results are difficult to compare.

### Mechanic Pressure Devices

Other studies have looked into the stress-reducing effects of mechanic devices applying a constant pressure. For example, HR and state anxiety were compared in a group of 23 healthy students when they self-administered deep-pressure touch during 15 min while they were sandwiched in an apparatus called “Hug'm” (for “hug machine”) and when they just lay in the apparatus without deep pressure (119) (see Figure 2G). State anxiety and HR were not different in the two conditions. There was a trend for a larger anxiety reduction in participants with high trait anxiety when the machine was “on” compared to “off” than in participants with low trait anxiety. Using a similar machine, but with the squeeze being applied laterally and a larger amount of pressure, 40 healthy students were asked to describe their experience in the so-called squeeze machine (120); 45% of them used terms such as “relaxing.” Furthermore, ratings of relaxation were collected from 18 participants following stationary pressure and fast and slow rhythmic pressure of 3 min each. Relaxation was rated as being highest for slowly pulsating and stationary pressure compared to fast pulsating pressure.

Very recently, a series of experiments in healthy volunteers (*N* = 78 in total) evaluated the effects of pulsating pressure delivered with a sleeve-like device. Oscillating low compression of 30 mmHg resulted in a subjective decrease of anxiety similar to that obtained by slow CT-targeted stroking (121). High compression of 65 mmHg did not have such an effect.

Whereas, such pressure machines may not be assigned any human qualities such as intention, this appears to be different for devices with human-like features such as language. In this case, the beneficial effects of machine touch might be modulated by the assumed intention behind the touch. This is indicated by a study in which 56 healthy participants, divided into four groups of 14 participants each, received touch from a robotic nurse that verbally either gave a warning before the touch or not and, in case of the warning, gave reasons for the touch (122). The robot used a spatula-like end effector that was covered with a towel for moving across the participants' arm (see Figure 2H). Before or after the touch, depending on the condition, the participants received verbal information by the robot that they were going to get cleaned or received a comforting statement (“Everything will be alright; you are doing well.”) Affective touch was rated as more arousing than instrumental touch. Positive and negative affect did not differ for the two touch types. Touch preceded by information was also rated as more arousing compared to when the information was given afterwards, and positive affect was lower for touch preceded by information. GSR increased following contact and during the touch in all four conditions, independent of whether the participants had received information before. At the same time, 10 out of 28 participants who had received comforting touch reported that they would have preferred if the robot had not touched them. In the group receiving instrumental touch, only one participant would have preferred no touch. This may point at the low acceptance of robotic touch which is explicitly performed with the attention to comfort. In the case of human touch, the assumed intention also determines touch perception and its effects (123, 124). On the other hand, the findings may have to do with the way the information was conveyed, as the authors themselves point out. More controlled experiments on such contextual effects are needed.

As with the animal studies, the low number of studies meeting our criteria and the different effect measures used (see also Supplementary Table 1) make it difficult to compare their results. However, altogether one may conclude that touch by various non-human agents either has no or a small calming effect, which might be modulated by the assumed intention of the agent. Presumably, the appearance of the robot also plays a role here, as a too-human-look of robots can also have an opposite, negative effect on the interaction partner. This has been described as the “uncanny valley” effect [e.g., Moore (125)], where a robot resembling a human almost, but not perfectly, induces feelings of unfamiliarity and eeriness.

## Current Technical Advances in Robotics

While in classical industrial robotics the situation of a robot touching a human was considered as an emergency and had to be avoided, new research fields of human–robot interaction (HRI) and human–robot collaboration have emerged, where robots are expected to work side by side and even interact with humans similarly to a social interaction between two humans. Therefore, it is of highest relevance to evaluate these new and innovative systems also in regard to their psychological effects.

Assistive robots can be classified into two main categories (126): On the one hand, there are rehabilitation robots like smart wheelchairs (127) and exoskeletons, which can perform, e.g., a movement of a paralyzed hand by moving the hand for the patient (128). There is a collaboration with the human, yet no cooperation (129). The robot touch lies in the realm of HRI, as robots only act on a human and there is, normally, no joint effort with a human [e.g., handshaking; Shiomi et al. (130)].

On the other hand, there are socially assistive robots that directly interact with humans (131). This category includes service robots that can perform tasks, like handing an object and also washing or feeding (132, 133), and companion-like robots (111, 112). Both kinds of socially assistive robots have physical contact with humans; therefore, their touch can be expected to have psychological effects, and could be designed to act calming.

In general, from a technical view, humans are often considered as non-deterministic factors (134), that is, systems with unpredictable outputs despite identical inputs. This makes the development of HRI systems highly challenging and requires interdisciplinary collaboration between robotics experts, cognitive scientists, and psychologists in order to make human behavior at least, to some amount, more predictable by determining regularities and defining preferences.

Until now, robotic touch has been mostly investigated in the context of social robotics with humanoid robots (see “Section Touch between humans and artificial object”) when the robot actively touched a human, but there are also a number of studies where the robot is touched by a human and responds with different forms of feedback (110–112). The feedback of the robot may be important for shaping the experience of the touch provider, as indicated by the results from the animated vs. non-animated Haptic Creature (112) and also from a living animal vs. a stuffed animal toy (108). When programming and building social robots, studies in which a robot is the toucher and a human user is the touchee are of high interest to determine the effects of different kinds of robotic touch on stress outcomes. Ideally, there are identifiable characteristics of the touch, the robot, and the situation, which allow specifying when robotic touch can be experienced as a safety signal and when not.

Although robotic touch is usually associated with humanoid robots (135), plain touch arousal is feasible with just a one degree-of-freedom (DOF) linear actuator (45, 136). In this basic research experiment with a machine without human appearance, the trajectory, the moving speed, and the contact force were predefined and had no variance. However, it is arguable if variations in trajectory parameters are beneficial for a natural feeling of repetitive touch. On the other hand, in the study of Willemse and van Erp (137), a humanoid robot “NAO” with 25 DOF, touch sensors, and cameras were used. This allowed for very different and sophisticated movements toward and pressure onto a person who might move herself. For the touch experiment, a teleoperation mode was utilized. The operator was initiating a social touch using one robot (master), while the unaware participants were touched on the shoulder by another robot (slave) connected to the first one. It is worth investigating whether these differentiated ways of touch influence its stress-reducing effects.

**Figure 3:**
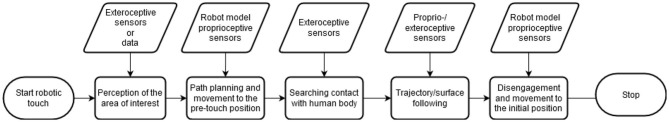
Flow chart of a simplified robotic touch. Steps 2 to 4 are expected to crucially influence the perception of touch and, therefore, its role as safety signal.

A simplified robotic touch process is similar to a grasping action and can be divided into the following steps (see Figure 3): (1) perception of the area of interest (e.g., human forearm), (2) path planning and end-effector movement from the initial position to the pre-touch position, (3) searching contact with human body, (4) trajectory/surface following, and (5) disengagement and moving back to the initial position. Whereas, the movement to the pre-touch position (step 2) can be realized with a simple position controller, an interaction control policy is necessary for the trajectory/surface tracking (step 4). A survey of interaction control schemes with static and dynamic model-based compensation is presented by Chiaverini et al. (138). Especially in steps 2–4, technical adjustments can be done to optimize the effects of touch. This indicates the complexity of parameters that has to be taken into account.

One step into this direction was the study of Reed and Peshkin (139), where the authors attempted a “Haptic Turing Test” in an experimental setup with a two-handled crank with a hidden motor. A Turing test assesses a machine's ability to exhibit intelligent behavior comparable to or even indistinguishable from human behavior. For the investigation of dyadic physical communication, the authors designed a simple task of mutually acquiring a one-DOF visual target. In one of the experiments, 10 out of 11 participants who worked with a hidden robot in the presence of a confederate were under the impression that they were working with another human. A “Touch Turing Test” can also be designed. The human visual and auditory systems provide powerful sensory input that complicates isolated touch experiments with humanoid or industrial robots visible to the participants. The Touch Turing Test with an autonomous robot executing variable touch motions in the presence of a confederate could help investigate the hypothesis on whether or not a robot is able to simulate human touch motion sufficiently well to deceive human participants—and whether deception is a good idea in this case. In addition, recent technological advances in virtual reality (VR) (140) offer a new modality for touch experiments. Humans can be touched by a robot in reality, and a digital twin in the form of a humanoid robot, robotic arm, or even a human could be shown to the participant in VR. Similar to the experiments with the “Repliee Q2” (141), the “uncanny valley” effect (see “Section Mechanic pressure devices”) can be investigated with addition of the tactile sense and underlining the relevance of taking the psychological context of touch into account. Studies making use of these possibilities could provide important insight into the optimal properties of robotic touch.

In an overview of interpersonal touch, Gallace and Spence (142) point out that surprisingly little systematic scientific research has been conducted on this topic. The characteristics of tactile stimulation that are needed for the touch to be perceived by a human as interpersonal rather than as mechanical are still unknown. Furthermore, in order to realize an autonomous robotic touch in the context of physical HRI, it is essential that the robot can use sensory feedback and perception. Besides the visual (e.g., RGB and depth cameras) and force-torque sensor technologies already widely used in HRI, tactile and proximity sensors could enhance the performance of the robotic touch system. Multi-modal tactile proximity sensors (143, 144) can be applied for the searching contact phase (step 3 of Figure 3) and during the trajectory tracking phase for the pressure/force feedback.

Finally, latest breakthroughs in artificial intelligence research can be employed both as a control policy model [e.g., reinforcement/machine learning (145)] and as a feedback to the robotic system using artificial emotional intelligence (146), therefore already allowing to adapt the kind of touch during the interaction. For instance, facial expression recognition can achieve very high accuracy (~97%) under laboratory conditions (147). Facial expressions or acoustic speech can be perceived by robot sensory systems and used as a feedback or reward signal for unsupervised learning. In a technically similar manner, a robotic system will be able to learn a personalized robotic touch based on the emotional feedback of the human participant and could account for individual preferences or clinical contexts. For example, the robot could adjust pressure based on the facial expression of the touch receiver. Such a dynamical adaptation to an individual's response would allow for greater flexibility and therefore presumably increase the likelihood of positive effects in the receiver.

Advances in robotics research as well as technological progress in hardware development bring robots from structured factory environments to human homes and enable new communication modalities for improved HRI. One of the important aspects in physical interaction and yet to become a growing research field in HRI is robotic touch, bringing robotics and psychology experts together.

## Conclusions and Clinical Implications

Taken together, the majority of previous research reviewed in this article indicates a calming/stress-relieving effect of touch, irrespective of the agent that is touching or being touched: effects have been shown both after actively touching, and being touched. Humans, animals, and even robotic devices may induce a cascade of reactions from tactile perception to insular control of the amygdala and subsequent regulation of the stress axes, resulting in dampened arousal. Both from onto- and phylogenetic perspectives, the health-related beneficial effects of physical interpersonal contact seem plausible, as they might signal safety from harm in the presence of the family or the community. Therefore, we propose that, under appropriate conditions, touch from various agents can act as social signal for safety and support mental and physical health. Clinical implications can be drawn for touch as a treatment for an acute physical or mental health problem under consideration of the disorder-specific reactions to touch and also for preventive applications during phases of high stress in order to reduce the chance of stress-induced diseases (148).

Interpersonal touch during medical treatments has been evaluated in a number of studies and suggests that touch can improve treatment outcomes [for a recent review, see (149)]. In their overview on the potentially beneficial effects of touch for patients treated in an intensive care unit, Harris et al. suggest that interpersonal touch is most effective when provided by a relative in a lightly moving way (in contrast to static touch) and that dimmed light might increase the calming effects. These conclusions are in line with research that light massage and touch can improve psychobiological stress levels and decrease pain in older patients with dementia (150) and patients with pain due to cancer (151). A touch therapy program in autistic children has been suggested to improve parent–child communication (152). Harris et al. (149) also support the notion that, rather than interpersonal touch *per se*, it is the affective level and the interpretation of an adequate physical contact as a gesture of support and closeness which can attenuate stress. This is in line with studies suggesting that some individuals (e.g., those who are anxious or traumatized) might prefer to have no physical contact to others or physical contact in a controlled setting only (19, 106). Above this, skin contact is associated with increased levels of intimacy and can bear the risk of re-traumatization after sexual trauma. Therefore, touch in a medical context (e.g., prior to surgery, during intensive care, or in a nursing home) should be well-elaborated in order to act beneficial. In line with this, in some vulnerable situations, being touched by a machine might be preferred over touch by an unknown person, such as when being washed. Another solution would be personalized touch that can be controlled by the individual's feedback itself.

Machine-based touch might also be helpful as a preventive procedure for lonely individuals or individuals in quarantine who cannot rely on human touch. This review article focuses on the basic research question of touch by different agents acting as safety signal; therefore, we did not discuss studies on infants or clinical patients in detail. However, this would, of course, be important for future applications of touch.

Expectations, beliefs, and the whole context of the interaction need to be taken into account, not only in clinical settings but in general, in order to determine the beneficial effects of touch [see (51)]. For example, sex/gender, and the romantic attraction to the interaction partner (153) have been found to influence the touch experience. The preference for physical contact with another human or an animal also differs between individuals (154–156) and is subject to personal experience such as trauma [e.g., Strauss et al. (19), Maier et al. (157)], touch deprivation (158), and attachment style (155).

Thus, various interindividual and context factors can also influence the effectiveness of touch as a safety signal. As stated by Older (159): “Appropriate touch becomes inappropriate when given at the wrong time, in the wrong dose, or to the wrong person.” Nevertheless, actual negative interaction situations have barely been investigated. Stress- or fear-dampening effects can only occur if the touch and the touching agent are not experienced as threatening or potentially dangerous. To the best of our knowledge, however, no study has yet focused on these different aspects of positive vs. negative anticipation of touch and the type and the quality of the relationship between the touching person or agent and the individual receiving the touch. Rather than the objective characteristics of the touch itself, it might be the interpretation and the social situation which makes touch either act as a safety signal or a threat. To disentangle these effects, research systematically testing different contexts, and expectations would be necessary. Nevertheless, when reviewing the literature, it becomes clear that this field of research is facing several challenges. In studies of human touch, there is a large heterogeneity of the way touch was performed or instructed. In ecologically more valid studies such as those regarding touch in close relationships and therapeutic touch, e.g., massages or animal-assisted therapy, the effects of touch may be intermixed with the effects of other experiences in this social interaction. In some studies, other aspects such as visual appearance or verbal communication are not controlled for or there is no control group at all. Therefore, the results have to be treated with caution in regard to the basic research questions on touch as a safety signal.

Regarding the stress- or fear-markers assessed, there is also a lot of variance. Cortisol, as an established marker of HPA activity (67), is the most widely used physiological measure, which improves comparability among studies, yet studies reporting other outcomes are difficult to integrate (see Supplementary Table 1 for an overview over the measures used). Another limitation of some studies is the critically small sample size that may hamper the generalization of the results. However, despite these limitations, the sum of research points toward similar effects, which is the regulation of the stress axes by touch. Future research needs to address the issues listed above to provide a clear picture on the physical conditions in which touch acts as stress-dampening and by which underlying neural pathway it affects the stress axes. Our recommendations are both to design systematic research studies comparing different kinds of touch and also use established measures in the field.

Technical advances also open new research innovative directions: advances in machine learning and artificial emotional intelligence will allow the development of feasible robotic systems, which can account for individual preferences in terms of personalized touch. Such touch by robots or machines might provide new options for individuals with an aversion for touch by another human [e.g., Hielscher and Mahar (154), Strauss et al. (19)].

In conclusion, the broad and multifaceted range of research on touch given by several agents can be summarized as a promising field, yet only at its very beginning. From a clinical perceptive though, robots or machines giving stress-relieving touch could be of high potential in healthcare, e.g., in patients in spatial isolation of quarantine, in individuals refusing touch by another person, in lonely people, or in nursery homes.

## Author Contributions

ME and US conceived the presented idea. ME led the review and the development of the manuscript, searched and summarized literature on human–human touch and on the neurobiological foundations of stress regulation. BD searched and summarized literature on touch in relationships. IM contributed with technical expertise and wrote on advances in the field of robotics. US searched and summarized literature on human–robot and human–animal studies. All authors have written and proofread the manuscript. ME and US revised the manuscript following the reviewers' comments.

## Conflict of Interest

The authors declare that the research was conducted in the absence of any commercial or financial relationships that could be construed as a potential conflict of interest.

## References

[1] McGlone F Wessberg J Olausson H. Discriminative and affective touch: sensing and feeling. Neuron. (2014) 82:737–55. 10.1016/j.neuron.2014.05.001 24853935

[2] Cascio CJ Moore D McGlone F. Social touch and human development. Dev Cogn Neurosci. (2019) 35:5–11. 10.1016/j.dcn.2018.04.009 29731417 PMC6968965

[3] Field T. Touch for socioemotional and physical well-being: a review. Dev Rev. (2010) 30:367–83. 10.1016/j.dr.2011.01.001

[4] Holt-Lunstad J Smith TB Baker M Harris T Stephenson D. Loneliness and social isolation as risk factors for mortality: a meta-analytic review. Perspect Psychol Sci. (2015) 10:227–37. 10.1177/1745691614568352 25910392

[5] Dunbar RI. The social role of touch in humans and primates: behavioural function and neurobiological mechanisms. Neurosci Biobehav Rev. (2010) 34:260–8. 10.1016/j.neubiorev.2008.07.001 18662717

[6] Hertenstein MJ Keltner D App B Bulleit BA Jaskolka AR. Touch communicates distinct emotions. Emotion. (2006) 6:528–33. 10.1037/1528-3542.6.3.528 16938094

[7] Hertenstein MJ Holmes R McCullough M Keltner D. The communication of emotion via touch. Emotion. (2009) 9:566–73. 10.1037/a0016108 19653781

[8] Hauser SC McIntyre S Israr A Olausson H Gerling GJ. Uncovering human-to-human physical interactions that underlie emotional and affective touch communication. In: *2019 IEEE World Haptics Conference, WHC*. Tokyo: IEEE (2019). p. 407–12. 10.1109/WHC.2019.8816169 PMC8419857 34493952

[9] Taira K Rolls ET. Receiving grooming as a reinforcer for the monkey. Physiol Behav. (1996) 59:1189–92. 10.1016/0031-9384(95)02213-9 8737912

[10] Pawling R Cannon PR McGlone FP Walker SC. C-tactile afferent stimulating touch carries a positive affective value. PLoS ONE. (2017) 12:e0173457. 10.1371/journal.pone.0173457 28282451 PMC5345811

[11] Burleson MH Davis MC. 10 social touch and resilience. In: Kent M, Davis MC, ReichRoutledge JW, editors. The Resilience Handbook: Approaches to Stress and Trauma. London: Routledge (2013). p. 131.

[12] Morrison I. Keep calm and cuddle on: social touch as a stress buffer. Adapt Hum Behav Physiol. (2016) 2:344–62. 10.1007/s40750-016-0052-x

[13] Ditzen B Neumann ID Bodenmann G von Dawans B Turner RA Ehlert U. Effects of different kinds of couple interaction on cortisol and heart rate responses to stress in women. Psychoneuroendocrinology. (2007) 32:565–74. 10.1016/j.psyneuen.2007.03.011 17499441

[14] Triscoli C Croy I Steudte-Schmiedgen S Olausson H Sailer U. Heart rate variability is enhanced by long-lasting pleasant touch at CT-optimized velocity. Biol Psychol. (2017) 128:71–81. 10.1016/j.biopsycho.2017.07.007 28723347

[15] Kreuder AK Wassermann L Wollseifer M Ditzen B Eckstein M Stoffel-Wagner B. Oxytocin enhances the pain-relieving effects of social support in romantic couples. Hum Brain Mapp. (2019) 40:242–51. 10.1002/hbm.24368 30152573 PMC6865468

[16] Scheele D Kendrick KM Khouri C Kretzer E Schläpfer TE Stoffel-Wagner B. An oxytocin-induced facilitation of neural and emotional responses to social touch correlates inversely with autism traits. Neuropsychopharmacology. (2014) 39:2078–85. 10.1038/npp.2014.78 24694924 PMC4104346

[17] Koyama T McHaffie JG Laurienti PJ Coghill RC. The subjective experience of pain: where expectations become reality. Proc Natl Acad Sci USA. (2005) 102:12950–5. 10.1073/pnas.0408576102 16150703 PMC1200254

[18] Floyd K. Relational and health correlates of affection deprivation. West J Commun. (2014) 78:383–403. 10.1080/10570314.2014.927071

[19] Strauss T Rottstadt F Sailer U Schellong J Hamilton JP Raue C. Touch aversion in patients with interpersonal traumatization. Depress Anxiety. (2019) 36:635–46. 10.1002/da.22914 31209965

[20] Phelps EA LeDoux JE. Contributions of the amygdala to emotion processing: from animal models to human behavior. Neuron. (2005) 48:175–87. 10.1016/j.neuron.2005.09.025 16242399

[21] Janak PH Tye KM. From circuits to behaviour in the amygdala. Nature. (2015) 517:284. 10.1038/nature14188 25592533 PMC4565157

[22] LeDoux JE. Emotion circuits in the brain. Ann Rev Neurosci. (2000) 23:155–84. 10.1146/annurev.neuro.23.1.155 10845062

[23] Ehrlich I Humeau Y Grenier F Ciocchi S Herry C Lüthi A. Amygdala inhibitory circuits and the control of fear memory. Neuron. (2009) 62:757–71. 10.1016/j.neuron.2009.05.026 19555645

[24] Amano T Duvarci S Popa D Paré D. The fear circuit revisited: contributions of the basal amygdala nuclei to conditioned fear. J Neurosci. (2011) 31:15481–9. 10.1523/JNEUROSCI.3410-11.2011 22031894 PMC3221940

[25] Milad MR Quirk GJ. Neurons in medial prefrontal cortex signal memory for fear extinction. Nature. (2002) 420:70. 10.1038/nature01138 12422216

[26] McDonald AJ Shammah-Lagnado SJ Shi C Davis M. Cortical afferents to the extended amygdala. Ann N Y Acad Sci. (1999) 877:309–38. 10.1111/j.1749-6632.1999.tb09275.x 10415657

[27] Foilb AR Flyer-Adams JG Maier SF Christianson JP. Posterior insular cortex is necessary for conditioned inhibition of fear. Neurobiol Learn Memory. (2016) 134:317–27. 10.1016/j.nlm.2016.08.004 27523750 PMC5424894

[28] Rodgers KM Benison AM Klein A Barth DS. Auditory, somatosensory, and multisensory insular cortex in the rat. Cereb Cortex. (2008) 18:2941–51. 10.1093/cercor/bhn054 18424777 PMC2583160

[29] Shi CJ Cassell M. Cascade projections from somatosensory cortex to the rat basolateral amygdala via the parietal insular cortex. J Comp Neurol. (1998) 399:469–91. 9741478 10.1002/(sici)1096-9861(19981005)399:4<469::aid-cne3>3.0.co;2-#

[30] Pavlov IP. Conditioned Reflexes: An Investigation of the Physiological Activity of the Cerebral Cortex. Anrep GV, editor. London: Oxford University Press (1927). 10.5214/ans.0972-7531.1017309 PMC4116985 25205891

[31] Öhman A Mineka S. Fears, phobias, and preparedness: toward an evolved module of fear and fear learning. Psychol Rev. (2001) 108:483–522. 10.1037/0033-295x.108.3.483 11488376

[32] Eisenberger NI Master SL Inagaki TK Taylor SE Shirinyan D Lieberman MD. Attachment figures activate a safety signal-related neural region and reduce pain experience. Proc Natl Acad Sci USA. (2011) 108:11721–6. 10.1073/pnas.1108239108 21709271 PMC3136329

[33] Kong E Monje FJ Hirsch J Pollak DD. Learning not to fear: neural correlates of learned safety. Neuropsychopharmacology. (2014) 39:515. 10.1038/npp.2013.191 23963118 PMC3895233

[34] Christianson JP Benison AM Jennings J Sandsmark EK Amat J Kaufman RD. The sensory insular cortex mediates the stress-buffering effects of safety signals but not behavioral control. J Neurosci. (2008) 28:13703–11. 10.1523/JNEUROSCI.4270-08.2008 19074043 PMC2667691

[35] Dickinson A Pearce JM. Inhibitory interactions between appetitive and aversive stimuli. Psychol Bull. (1977) 84:690. 10.1037/0033-2909.84.4.690

[36] Pollak DD Monje FJ Zuckerman L Denny CA Drew MR Kandel ER. An animal model of a behavioral intervention for depression. Neuron. (2008) 60:149–61. 10.1016/j.neuron.2008.07.041 18940595 PMC3417703

[37] Rogan MT Leon KS Perez DL Kandel ER. Distinct neural signatures for safety and danger in the amygdala and striatum of the mouse. Neuron. (2005) 46:309–20. 10.1016/j.neuron.2005.02.017 15848808

[38] Pollak DD Rogan MT Egner T Perez DL Yanagihara TK Hirsch J. A translational bridge between mouse and human models of learned safety. Ann Med. (2010) 42:127–34. 10.3109/07853890903583666 20121549

[39] Belujon P Grace AA. Regulation of dopamine system responsivity and its adaptive and pathological response to stress. Proc R Soc B Biol Sci. (2015) 282:20142516. 10.1098/rspb.2014.2516 25788601 PMC4389605

[40] Dutcher JM Creswell JD. The role of brain reward pathways in stress resilience and health. Neurosci Biobehav Rev. (2018) 95:559–67. 10.1016/j.neubiorev.2018.10.014 30477985

[41] McCabe C Rolls ET Bilderbeck A McGlone F. Cognitive influences on the affective representation of touch and the sight of touch in the human brain. Soc Cogn Affect Neurosci. (2008) 3:97–108. 10.1093/scan/nsn005 19015100 PMC2555465

[42] Rolls ET. The affective and cognitive processing of touch, oral texture, and temperature in the brain. Neurosci Biobehav Rev. (2010) 34:237–45. 10.1016/j.neubiorev.2008.03.010 18468687

[43] Lindgren L Westling G Brulin C Lehtipalo S Andersson M Nyberg L. Pleasant human touch is represented in pregenual anterior cingulate cortex. Neuroimage. (2012) 59:3427–32. 10.1016/j.neuroimage.2011.11.013 22100768

[44] Ackerley R Backlund Wasling H Liljencrantz J Olausson H Johnson RD Wessberg J. Human C-tactile afferents are tuned to the temperature of a skin-stroking caress. J Neurosci. (2014) 34:2879–83. 10.1523/JNEUROSCI.2847-13.2014 24553929 PMC3931502

[45] Morrison I Löken LS Olausson H. The skin as a social organ. Exp Brain Res. (2010) 204:305–14. 10.1007/s00221-009-2007-y 19771420

[46] Olausson H Lamarre Y Backlund H Morin C Wallin B Starck G. Unmyelinated tactile afferents signal touch and project to insular cortex. Nat Neurosci. (2002) 5:900. 10.1038/nn896 12145636

[47] Morrison I Löken LS Minde J Wessberg J Perini I Nennesmo I. Reduced C-afferent fibre density affects perceived pleasantness and empathy for touch. Brain. (2011) 134:1116–26. 10.1093/brain/awr011 21378097

[48] Gordon I Voos AC Bennett RH Bolling DZ Pelphrey KA Kaiser MD. Brain mechanisms for processing affective touch. Hum Brain Mapp. (2013) 34:914–22. 10.1002/hbm.21480 22125232 PMC6869848

[49] Kirsch LP Besharati S Papadaki C Crucianelli L Bertagnoli S Ward N. Damage to the right insula disrupts the perception of affective touch. eLife. (2020) 9:e47895 10.7554/eLife.47895 31975686 PMC7043887

[50] Eriksson Hagberg E Ackerley R Lundqvist D Schneiderman J Jousmäki V Wessberg J. Spatio-temporal profile of brain activity during gentle touch investigated with magnetoencephalography. NeuroImage. (2019) 201:116024 10.1016/j.neuroimage.2019.116024 31323258

[51] Ellingsen D-M Leknes S Løseth G Wessberg J Olausson H. The neurobiology shaping affective touch: expectation, motivation, and meaning in the multisensory context. Front Psychol. (2016) 6:1986. 10.3389/fpsyg.2015.01986 26779092 PMC4701942

[52] May AC Stewart JL Paulus MP Tapert SF. The effect of age on neural processing of pleasant soft touch stimuli. Front Behav Neurosci. (2014) 8:52. 10.3389/fnbeh.2014.00052 24600366 PMC3930859

[53] Sailer U Triscoli C Häggblad G Hamilton P Olausson H Croy I. Temporal dynamics of brain activation during 40 minutes of pleasant touch. NeuroImage. (2016) 139:360–7. 10.1016/j.neuroimage.2016.06.031 27338514

[54] Uvnäs-Moberg K Handlin L Petersson M. Self-soothing behaviors with particular reference to oxytocin release induced by non-noxious sensory stimulation. Front Psychol. (2015) 5:1529. 10.3389/fpsyg.2014.01529 25628581 PMC4290532

[55] Walker SC Trotter PD Swaney WT Marshall A McGlone FP. C-tactile afferents: cutaneous mediators of oxytocin release during affiliative tactile interactions? Neuropeptides. (2017) 64:27–38. 10.1016/j.npep.2017.01.001 28162847

[56] Ditzen B Eckstein M Fischer M Aguilar-Raab C. Partnerschaft und gesundheit. Psychotherapeut. (2019) 64:482–8. 10.1007/s00278-019-00379-9

[57] Holt-Lunstad J Birmingham W Light KC. The influence of depressive symptomatology and perceived stress on plasma and salivary oxytocin before, during and after a support enhancement intervention. Psychoneuroendocrinology. (2011) 36:1249–56. 10.1016/j.psyneuen.2011.03.007 21507578

[58] de Jong TR Menon R Bludau A Grund T Biermeier V Klampfl SM. Salivary oxytocin concentrations in response to running, sexual self-stimulation, breastfeeding and the TSST: the regensburg oxytocin challenge (ROC) study. Psychoneuroendocrinology. (2015) 62:381–8. 10.1016/j.psyneuen.2015.08.027 26385109

[59] Portnova GV Proskurnina EV Sokolova SV Skorokhodov IV Varlamov AA. Perceived pleasantness of gentle touch in healthy individuals is related to salivary oxytocin response and EEG markers of arousal. Exp Brain Res. (2020) 238:2257–68. 10.1007/s00221-020-05891-y 32719908

[60] Tang Y Benusiglio D Lefevre A Hilfiger L Althammer F Bludau A. Social touch promotes interfemale communication via activation of parvocellular oxytocin neurons. Nat Neurosci. (2020) 23:1125–37. 10.1038/s41593-020-0674-y 32719563

[61] Grewen KM Light KC. Plasma oxytocin is related to lower cardiovascular and sympathetic reactivity to stress. Biol Psychol. (2011) 87:340–9. 10.1016/j.biopsycho.2011.04.003 21540072 PMC3225916

[62] Eckstein M Becker B Scheele D Scholz C Preckel K Schlaepfer TE. Oxytocin facilitates the extinction of conditioned fear in humans. Biol Psychiatry. (2015) 78:194–202. 10.1016/j.biopsych.2014.10.015 25542304

[63] Boccia M Petrusz P Suzuki K Marson L Pedersen C. Immunohistochemical localization of oxytocin receptors in human brain. Neuroscience. (2013) 253:155–64. 10.1016/j.neuroscience.2013.08.048 24012742

[64] Sapolsky RM Romero LM Munck AU. How do glucocorticoids influence stress responses? Integrating permissive, suppressive, stimulatory, and preparative actions. Endocr Rev. (2000) 21:55–89. 10.1210/er.21.1.55 10696570

[65] Godoy LD Rossignoli MT Delfino-Pereira P Garcia-Cairasco N de Lima Umeoka EH. A comprehensive overview on stress neurobiology: basic concepts and clinical implications. Front Behav Neurosci. (2018) 12:127. 10.3389/fnbeh.2018.00127 30034327 PMC6043787

[66] McEwen BS. The neurobiology of stress: from serendipity to clinical relevance. Brain Res. (2000) 886:172–89. 10.1016/S0006-8993(00)02950-4 11119695

[67] Kirschbaum C Hellhammer DH. Salivary cortisol in psychoneuroendocrine research: recent developments and applications. Psychoneuroendocrinology. (1994) 19:313–33. 10.1016/0306-4530(94)90013-2 8047637

[68] Nater UM Rohleder N. Salivary alpha-amylase as a non-invasive biomarker for the sympathetic nervous system: current state of research. Psychoneuroendocrinology. (2009) 34:486–96. 10.1016/j.psyneuen.2009.01.014 19249160

[69] Thayer JF Åhs F Fredrikson M Sollers JJ Wager TD. A meta-analysis of heart rate variability and neuroimaging studies: implications for heart rate variability as a marker of stress and health. Neurosci Biobehav Rev. (2012) 36:747–56. 10.1016/j.neubiorev.2011.11.009 22178086

[70] Lee Masson H Op de Beeck H. Socio-affective touch expression database. PLoS ONE. (2018) 13:e0190921. 10.1371/journal.pone.0190921 29364988 PMC5783378

[71] Kawamichi H Kitada R Yoshihara K Takahashi HK Sadato N. Interpersonal touch suppresses visual processing of aversive stimuli. Front Hum Neurosci. (2015) 9:164. 10.3389/fnhum.2015.00164 25904856 PMC4389358

[72] Ditzen B Germann J Meuwly N Bradbury TN Bodenmann G Heinrichs M. Intimacy as related to cortisol reactivity and recovery in couples undergoing psychosocial stress. Psychosom Med. (2019) 81:16–25. 10.1097/PSY.0000000000000633 30134358

[73] Ditzen B Hoppmann C Klumb P. Positive couple interactions and daily cortisol: on the stress-protecting role of intimacy. Psychosom Med. (2008) 70:883–9. 10.1097/PSY.0b013e318185c4fc 18842747

[74] Debrot A Schoebi D Perrez M Horn AB. Touch as an interpersonal emotion regulation process in couples' daily lives: the mediating role of psychological intimacy. Pers Soc Psychol Bull. (2013) 39:1373–85. 10.1177/0146167213497592 23885034

[75] Light KC Grewen KM Amico JA. More frequent partner hugs and higher oxytocin levels are linked to lower blood pressure and heart rate in premenopausal women. Biol Psychol. (2005) 69:5–21. 10.1016/j.biopsycho.2004.11.002 15740822

[76] Floyd K Mikkelson AC Tafoya MA Farinelli L La Valley AG Judd J. Human affection exchange: XIV. relational affection predicts resting heart rate and free cortisol secretion during acute stress. Behav Med. (2007) 32:151–6. 10.3200/BMED.32.4.151-156 17348430

[77] Coan JA Schaefer HS Davidson RJ. Lending a hand: Social regulation of the neural response to threat. Psychol Sci. (2006) 17:1032–9. 10.1111/j.1467-9280.2006.01832.x 17201784

[78] Graff TC Luke SG Birmingham WC. Supportive hand-holding attenuates pupillary responses to stress in adult couples. PLoS ONE. (2019) 14:e0212703. 10.1371/journal.pone.0212703 30794665 PMC6386442

[79] Grewen KM Anderson BJ Girdler SS Light KC. Warm partner contact is related to lower cardiovascular reactivity. Behav Med. (2003) 29:123–30. 10.1080/08964280309596065 15206831

[80] Mercado E Hibel L. I love you from the bottom of my hypothalamus: the role of stress physiology in romantic pair bond formation and maintenance. Soc Personal Psychol Compass. (2017) 11:e12298. 10.1111/spc3.12298 30220909 PMC6135532

[81] Sripongngam T Eungpinichpong W Sirivongs D Kanpittaya J Tangvoraphonkchai K Chanaboon S. Immediate effects of traditional Thai massage on psychological stress as indicated by salivary alpha-amylase levels in healthy persons. Med Science Monitor Basic Res. (2015) 21:216. 10.12659/MSMBR.894343 26436433 PMC4599180

[82] Drescher VM Gantt WH Whitehead WE. Heart rate response to touch. Psychosom Med. (1980). 42:559–65. 10.1097/00006842-198011000-00004 7465741

[83] Drescher VM Whitehead WE Morrill-Corbin ED Cataldo MF. Physiological and subjective reactions to being touched. Psychophysiology. (1985) 22:96–100. 10.1111/j.1469-8986.1985.tb01565.x 3975324

[84] Pawling R Trotter PD McGlone FP Walker SC. A positive touch: C-tactile afferent targeted skin stimulation carries an appetitive motivational value. Biol Psychol. (2017) 129:186–94. 10.1016/j.biopsycho.2017.08.057 28865933

[85] von Mohr M Krahe C Beck B Fotopoulou A. The social buffering of pain by affective touch: a laser-evoked potential study in romantic couples. Soc Cogn Affect Neurosci. (2018) 13:1121–30. 10.1093/scan/nsy085 30247679 PMC6234321

[86] von Mohr M Kirsch LP Fotopoulou A. The soothing function of touch: affective touch reduces feelings of social exclusion. Sci Rep. (2017) 7:13516. 10.1038/s41598-017-13355-7 29044137 PMC5647341

[87] Weiss SJ. Effects of differential touch on nervous system arousal of patients recovering from cardiac disease. Heart Lung. (1990) 19 (5 Pt. 1):474–80. 2170296

[88] Maratos FA Duarte J Barnes C McEwan K Sheffield D Gilbert P. The physiological and emotional effects of touch: Assessing a hand-massage intervention with high self-critics. Psychiatry Res. (2017) 250:221–7. 10.1016/j.psychres.2017.01.066 28167436

[89] Kirschner M Kirschner R. Hand massage reduces perceived stress, anxiety and fatigue. Int J Innov Stud Med Sci. (2019) 3.

[90] Osaka I Kurihara Y Tanaka K Nishizaki H Aoki S Adachi I. Endocrinological evaluations of brief hand massages in palliative care. J Altern Complement Med. (2009) 15:981–5. 10.1089/acm.2008.0241 19757975

[91] Brand LR Munroe DJ Gavin J. The effect of hand massage on preoperative anxiety in ambulatory surgery patients. AORN J. (2013) 97:708–17. 10.1016/j.aorn.2013.04.003 23722035

[92] Listing M Krohn M Liezmann C Kim I Reisshauer A Peters E. The efficacy of classical massage on stress perception and cortisol following primary treatment of breast cancer. Arch Women's Mental Health. (2010) 13:165–73. 10.1007/s00737-009-0143-9 20169378

[93] Hernandez-reif M Field T Krasnegor J Theakston H. Lower back pain is reduced and range of motion increased after massage therapy. Int J Neurosci. (2001) 106:131–45. 10.3109/00207450109149744 11264915

[94] Field TM Hernandez-Reif M Quintino O Schanberg S Kuhn C. Elder retired volunteers benefit from giving massage therapy to infants. J Appl Gerontol. (1998) 17:229–39. 10.1177/073346489801700210

[95] Edwards DJ Young H Johnston R. The immediate effect of therapeutic touch and deep touch pressure on range of motion, interoceptive accuracy and heart rate variability: a randomized controlled trial with moderation analysis. Front Integrat Neurosci. 12:41. 10.3389/fnint.2018.00041 PMC6160827 30297988

[96] Lindgren L Rundgren S Wins ö O Lehtipalo S Wiklund U Karlsson M. (2010). Physiological responses to touch massage in healthy volunteers. Autonom Neurosci. 158:105–10. 10.1016/j.autneu.2010.06.011 20638912

[97] Diego MA Field T. Moderate pressure massage elicits a parasympathetic nervous system response. Int J Neurosci. (2009) 119:630–8. 10.1080/00207450802329605 19283590

[98] Charry-Sánchez JD Pradilla I Talero-Gutiérrez C. Animal-assisted therapy in adults: a systematic review. Complement Ther Clin Pract. (2018) 32:169–80. 10.1016/j.ctcp.2018.06.011 30057046

[99] Vormbrock JK Grossberg JM. Cardiovascular effects of human-pet dog interactions. J Behav Med. (1988) 11:509–17. 10.1007/BF00844843 3236382

[100] Handlin L Hydbring-Sandberg E Nilsson A Ejdebäck M Jansson A Uvnäs-Moberg K. Short-term interaction between dogs and their owners: effects on oxytocin, cortisol, insulin and heart rate—an exploratory study. Anthrozoös. (2011) 24:301–15. 10.2752/175303711X13045914865385

[101] Hama H Yogo M Matsuyama Y. Effects of stroking horses on both humans' and horses' heart rate responses. Japan Psychol Res. (1996) 38:66–73. 10.1111/j.1468-5884.1996.tb00009.x

[102] Baun MM Bergstrom N Langston NF Thoma L. Physiological effects of human/companion animal bonding. Nurs Res. (1984) 33:126–9. 10.1097/00006199-198405000-00002 6563527

[103] Jenkins JL. Physiological effects of petting a companion animal. Psychol Rep. (1986) 58:21–2. 10.2466/pr0.1986.58.1.21 3961065

[104] Wilson CC. Physiological responses of college students to a pet. J Nerv Mental Dis. (1987) 175:606–12. 10.1097/00005053-198710000-00005 3655768

[105] Odendaal JSJ Meintjes RA. Neurophysiological correlates of affiliative behaviour between humans and dogs. Vet J. (2003) 165:296–301. 10.1016/S1090-0233(02)00237-X 12672376

[106] Wheeler EA Faulkner ME. The pet effect physiological calming in the presence of canines. Soc Anim. (2015) 23:425–38. 10.1163/15685306-12341374

[107] Kirschbaum C Pirke K-M Hellhammer DH. The ‘trier social stress test'–a tool for investigating psychobiological stress responses in a laboratory setting. Neuropsychobiology. (1993) 28:76–81. 10.1159/000119004 8255414

[108] Shiloh S Sorek G Terkel J. Reduction of state-anxiety by petting animals in a controlled laboratory experiment. Anxiety Stress Coping. (2003) 16:387–95. 10.1080/1061580031000091582

[109] Straatman I Hanson EKS Endenburg N Mol JA. The influence of a dog on male students during a stressor. Anthrozoös. (1997) 10:191–7. 10.2752/089279397787001012

[110] Robinson H MacDonald B Broadbent E. Physiological effects of a companion robot on blood pressure of older people in residential care facility: a pilot study. Australas J Ageing. (2015) 34:27–32. 10.1111/ajag.12099 24373064

[111] Shibata T Tanie K. Influence of a priori knowledge in subjective interpretation and evaluation by short-term interaction with mental commit robot. In: *Proceedings. 2000 IEEE/RSJ International Conference on Intelligent Robots and Systems*. Takamatsu: IEEE (2000). p. 169–74.

[112] Sefidgar YS MacLean KE Yohanan S Van der Loos HFM Croft EA Garland EJ. Design and evaluation of a touch-centered calming interaction with a social robot. IEEE Trans Affect Comp. (2016) 7:108–21. 10.1109/TAFFC.2015.2457893

[113] Willemse CJ van Erp JB. Social touch in human-robot interaction: Robot-initiated touches can induce positive responses without extensive prior bonding. Int J Soc Robot. (2019) 11:285–304.

[114] Willemse CJAM Toet A van Erp JBF. Affective and behavioral responses to robot-initiated social touch: toward understanding the opportunities and limitations of physical contact in human–robot interaction. Front ICT. (2017) 4:12. 10.3389/fict.2017.00012

[115] Cabibihan J Chauhan SS. Physiological responses to affective tele-touch during induced emotional stimuli. IEEE Trans Affect Comp. (2017) 8:108–18. 10.1109/TAFFC.2015.2509985

[116] Sumioka H Nakae A Kanai R Ishiguro H. Huggable communication medium decreases cortisol levels. Sci Rep. (2013) 3:3034. 10.1038/srep03034 24150186 PMC3805974

[117] Yamazaki R Christensen L Skov K Chang C-C Damholdt MF Sumioka H. Intimacy in phone conversations: anxiety reduction for danish seniors with hugvie. Front Psychol. (2016) 7:537–537. 10.3389/fpsyg.2016.00537 27148144 PMC4835483

[118] Keshmiri S Sumioka H Nakanishi J Ishiguro H. Bodily-contact communication medium induces relaxed mode of brain activity while increasing its dynamical complexity: a pilot study. Front Psychol. (2018) 9:1192. 10.3389/fpsyg.2018.01192 30050488 PMC6052895

[119] Krauss KE. The effects of deep pressure touch on anxiety. Am J Occup Ther. (1987) 41:366–73. 10.5014/ajot.41.6.366 3688151

[120] Grandin T. Calming effects of deep touch pressure in patients with autistic disorder, college students, and animals. J Child Adolesc Psychopharmacol. (1992) 2:63–72. 10.1089/cap.1992.2.63 19630623

[121] Case LK Liljencrantz J McCall MV Bradson M Necaise A Tubbs J. Pleasant deep pressure: expanding the social touch hypothesis. Neuroscience. (2020). 10.1016/j.neuroscience.2020.07.050 32768616 PMC7865002

[122] Chen TL King CHA Thomaz AL Kemp CC. An investigation of responses to robot-initiated touch in a nursing context. Int J Soc Robot. (2014) 6:141–61. 10.1007/s12369-013-0215-x

[123] Jakubiak BK Feeney BC. Affectionate touch to promote relational, psychological, and physical well-being in adulthood: a theoretical model and review of the research. Pers Soc Psychol Rev. (2017) 21:228–52. 10.1177/1088868316650307 27225036

[124] Rosenberger LA Ree A Eisenegger C Sailer U. Slow touch targeting CT-fibres does not increase prosocial behaviour in economic laboratory tasks. Sci Rep. (2018) 8:7700. 10.1038/s41598-018-25601-7 29769551 PMC5955966

[125] Moore RK. A bayesian explanation of the ‘uncanny valley'effect and related psychological phenomena. Sci Rep. (2012) 2:864. 10.1038/srep00864 23162690 PMC3499759

[126] Broekens J Heerink M Rosendal H. Assistive social robots in elderly care: a review. Gerontechnology. (2009) 8:94–103. 10.4017/gt.2009.08.02.002.00

[127] Desai S Mantha S Phalle V. Advances in smart wheelchair technology. In: *2017 International Conference on Nascent Technologies in Engineering, (ICNTE)*. Navi Mumbai: IEEE (2017). p. 1–7.

[128] Raab K Krakow K Tripp F Jung M. Effects of training with the ReWalk exoskeleton on quality of life in incomplete spinal cord injury: a single case study. Spinal Cord Ser Cases. (2016) 2:16019. 10.1038/scsandc.2015.25 28053728 PMC5125066

[129] Grosz B Kraus S. Collaborative plans for complex group action. Artif Intel. (1996) 86:269–357. 10.1016/0004-3702(95)00103-4

[130] Shiomi M Kanda T Ishiguro H Hagita N. Interactive humanoid robots for a science museum. In: P*roceedings of the 1st ACM SIGCHI/SIGART Conference on Human-Robot Interaction*. Salt Lake City, UT (2006). p. 305–12.

[131] Šabanović S Bennett CC Piatt JA Chang W Hakken D Kang S. Participatory design of socially assistive robots for preventive patient-centered healthcare. In: *IEEE/RSJ IROS Workshop on Assistive Robotics for Individuals With Disabilities* (2014).

[132] Graf B Reiser U Hägele M Mauz K Klein P. Robotic home assistant Care-O-bot® 3-product vision and innovation platform. In: *2009 IEEE Workshop on Advanced Robotics and its Social Impacts*. Tokyo: IEEE (2009). p. 139–44.

[133] Jacobs T Graf B. Practical evaluation of service robots for support and routine tasks in an elderly care facility. In: *2012 IEEE Workshop on Advanced Robotics and its Social Impacts*. (ARSO): Munich: IEEE (2012). p. 46–9.

[134] Bauer A Wollherr D Buss M. Human–robot collaboration: a survey. Int J. Human Robot. (2008) 5:47–66. 10.1142/S0219843608001303

[135] Kanda T Ishiguro H Ono T Imai M Nakatsu R. Development and evaluation of an interactive humanoid robot Robovie. In: *Proceedings 2002 IEEE International Conference on Robotics and Automation*. Washington, DC: IEEE (2002). p. 1848–55.

[136] McGlone F Vallbo AB Olausson H Loken L Wessberg J. Discriminative touch and emotional touch. Canad J Exp Psychol. (2007) 61:173. 10.1037/cjep2007019 17974312

[137] Willemse C van Erp JBF. Social touch in human-robot interaction: robot-initiated touches can induce positive responses without extensive prior bonding. Int J Soc Robot. (2019) 11:285–304. 10.1007/s12369-018-0500-9

[138] Chiaverini S Siciliano B Villani L. A survey of robot interaction control schemes with experimental comparison. IEEE/ASME Trans Mechatronic. (1999) 4:273–85. 10.1109/3516.789685

[139] Reed KB Peshkin MA. Physical collaboration of human-human and human-robot teams. IEEE Trans Haptics. (2008) 1:108–20. 10.1109/TOH.2008.13 27788067

[140] Anthes C García-Hernández RJ Wiedemann M Kranzlmüller D. State of the art of virtual reality technology. In: *2016 IEEE Aerospace Conference: IEEE*. Big Sky, MT (2016). p. 1–19.

[141] Tinwell A. The Uncanny Valley in Games and Animation. Boca Raton, FL: CRC Press (2014).

[142] Gallace A Spence C. The science of interpersonal touch: an overview. Neurosci Biobehav Rev. (2010) 34:246–59. 10.1016/j.neubiorev.2008.10.004 18992276

[143] Alagi H Navarro SE Mende M Hein B. A versatile and modular capacitive tactile proximity sensor. In: *2016 IEEE Haptics Symposium (HAPTICS)*. Philadelphia, PA: IEEE (2016). p. 290–6.

[144] Göger D Alagi H Wörn H. Tactile proximity sensors for robotic applications. In: *2013 IEEE International Conference on Industrial Technology (ICIT)*. Cape Town: IEEE (2013). p. 978–83.

[145] Arulkumaran K Deisenroth MP Brundage M Bharath AA. A brief survey of deep reinforcement learning. arXiv. (2017). 10.1109/MSP.2017.2743240

[146] Schuller D Schuller BW. The age of artificial emotional intelligence. Computer. (2018) 51:38–46. 10.1109/MC.2018.3620963

[147] Samadiani N Huang G Cai B Luo W Chi C-H Xiang Y. A review on automatic facial expression recognition systems assisted by multimodal sensor data. Sensors. (2019) 19:1863. 10.3390/s19081863 31003522 PMC6514576

[148] Ditzen B Heinrichs M. Psychobiology of social support: the social dimension of stress buffering. Restor Neurol Neurosci. (2014) 32:149–62. 10.3233/RNN-139008 23603443

[149] Harris SJ Papathanassoglou EDE Gee M Hampshaw SM Lindgren L Haywood A. Interpersonal touch interventions for patients in intensive care: a design-oriented realist review. Nurs Open. (2019) 6:216–35. 10.1002/nop2.200 30918674 PMC6419112

[150] Anderson AR Deng J Anthony RS Atalla SA Monroe TB. Using complementary and alternative medicine to treat pain and agitation in dementia: a review of randomized controlled trials from long-term care with potential use in critical care. Crit Care Nurs Clin North Am. (2017) 29:519–37. 10.1016/j.cnc.2017.08.010 29107312 PMC5687304

[151] Maindet C Burnod A Minello C George B Allano G Lemaire A. Strategies of complementary and integrative therapies in cancer-related pain-attaining exhaustive cancer pain management. Support Care Cancer. (2019) 27:3119–32. 10.1007/s00520-019-04829-7 31076901

[152] Escalona A Field T Singer-Strunck R Cullen C Hartshorn K. Brief report: improvements in the behavior of children with autism following massage therapy. J Autism Dev Disord. (2001) 31:513–6. 10.1023/A:1012273110194 11794416

[153] Gazzola V Spezio ML Etzel JA Castelli F Adolphs R Keysers C. Primary somatosensory cortex discriminates affective significance in social touch. Proc Natl Acad Sci USA. (2012) 109:E1657–66. 10.1073/pnas.1113211109 22665808 PMC3382530

[154] Hielscher E Mahar D. An exploration of the interaction between touch avoidance and the pleasant touch. (C-tactile afferent) system. Perception. (2016) 46:18–30. 10.1177/0301006616661938 27507262

[155] Krahé C von Mohr M Gentsch A Guy L Vari C Nolte T. Sensitivity to CT-optimal, affective touch depends on adult attachment style. Sci Rep. (2018) 8:14544. 10.1038/s41598-018-32865-6 30266979 PMC6162325

[156] Lundqvist L-O. Hyper-responsiveness to touch mediates social dysfunction in adults with autism spectrum disorders. Res Autism Spectr Disord. (2015) 9:13–20. 10.1016/j.rasd.2014.09.012

[157] Maier A Gieling C Heinen-Ludwig L Stefan V Schultz J Güntürkün O. Association of childhood maltreatment with interpersonal distance and social touch preferences in adulthood. Am J Psychiatry. (2020) 177:37–46. 10.1176/appi.ajp.2019.19020212 31416339

[158] Sailer U Ackerley R. Exposure shapes the perception of affective touch. Dev Cogn Neurosci. (2019) 35:109–14. 10.1016/j.dcn.2017.07.008 28818429 PMC6969125

[159] Older J. Touching is Healing: A Revolutionary Breakthrough in Medicine. New York, NY: Stein and Day. (1982).

